# Intraconal Retro-Orbital Cavernous Hemangioma Managed by Endoscopic Transnasal Excision: A Case Report

**DOI:** 10.7759/cureus.108240

**Published:** 2026-05-04

**Authors:** Archana Singh, Anil Kumar, Somen Misra

**Affiliations:** 1 Ophthalmology, All India Institute of Medical Sciences, Raipur, Raipur, IND; 2 Neurosurgery, All India Institute of Medical Sciences, Raipur, Raipur, IND

**Keywords:** endoscopic transnasal approach, intraconal tumor, minimally invasive orbital surgery, orbital cavernous hemangioma, orbital surgery, proptosis, retro-orbital mass

## Abstract

Cavernous hemangioma is the most common benign vascular lesion of the adult orbit, typically located in the intraconal compartment. Extraconal and medial locations are rare. We report the case of a 59-year-old male presenting with a 10-year history of progressive right eye proptosis associated with diminution of vision. Examination revealed abaxial (down and out) proptosis with mild restriction of ocular motility. Magnetic resonance imaging of the orbit showed a well-defined intraconal retrobulbar mass compressing the optic nerve. The lesion was completely excised via an endoscopic transnasal approach under general anesthesia. Intraoperatively, a vascular, encapsulated intraconal mass was identified beneath the medial and inferior rectus muscles and removed in toto. Histopathology confirmed cavernous hemangioma. Postoperatively, proptosis reduced from 26 mm to 22 mm, and final visual acuity improved from hand movements close to the face to 6/18. The suboptimal recovery in vision was due to foveal thinning owing to the compressive effect of the mass over the years. This case highlights the efficacy of the endoscopic transnasal approach in managing medially located intraconal cavernous hemangiomas. This approach provides excellent visualization, minimal morbidity, and superior cosmetic outcomes.

## Introduction

Cavernous hemangiomas are the most common vascular lesions of the orbit in adults, typically occurring in middle age with a female predominance [1]. They are often asymptomatic but may present with proptosis or decreased vision due to compression of the optic nerve [2]. Histologically, these lesions arise from the proliferation of vascular channels and hyperplasia of the vessel wall components.

More than 80% of orbital cavernous hemangiomas are located within the intraconal compartment, most commonly in the lateral aspect [3]. Extraconal and medial locations are uncommon [4]. Progressive, painless, axial proptosis is the usual mode of presentation [5]. However, in our case, the location of the lesion was medial in the intraconal compartment of the retrobulbar region, causing abaxial proptosis, which is a rare presentation. Surgical excision remains the mainstay of treatment, as these tumors are well-encapsulated and amenable to complete removal.

We report an unusual case of medial intraconal retro-orbital cavernous hemangioma presenting with abaxial proptosis, managed successfully via an endoscopic transnasal approach.

## Case presentation

A 59-year-old male presented to the ophthalmology outpatient department with complaints of progressive protrusion of the right eye (RE) associated with diminution of vision for 10-12 years. There was no history of pain, headache, diplopia, or nasal symptoms. There were no complaints of a sudden decrease in vision or a very rapid progression in the amount of proptosis. Hence, the possibility of a hemorrhagic, inflammatory, metastatic, or malignant mass could be ruled out.

**Figure 1:**
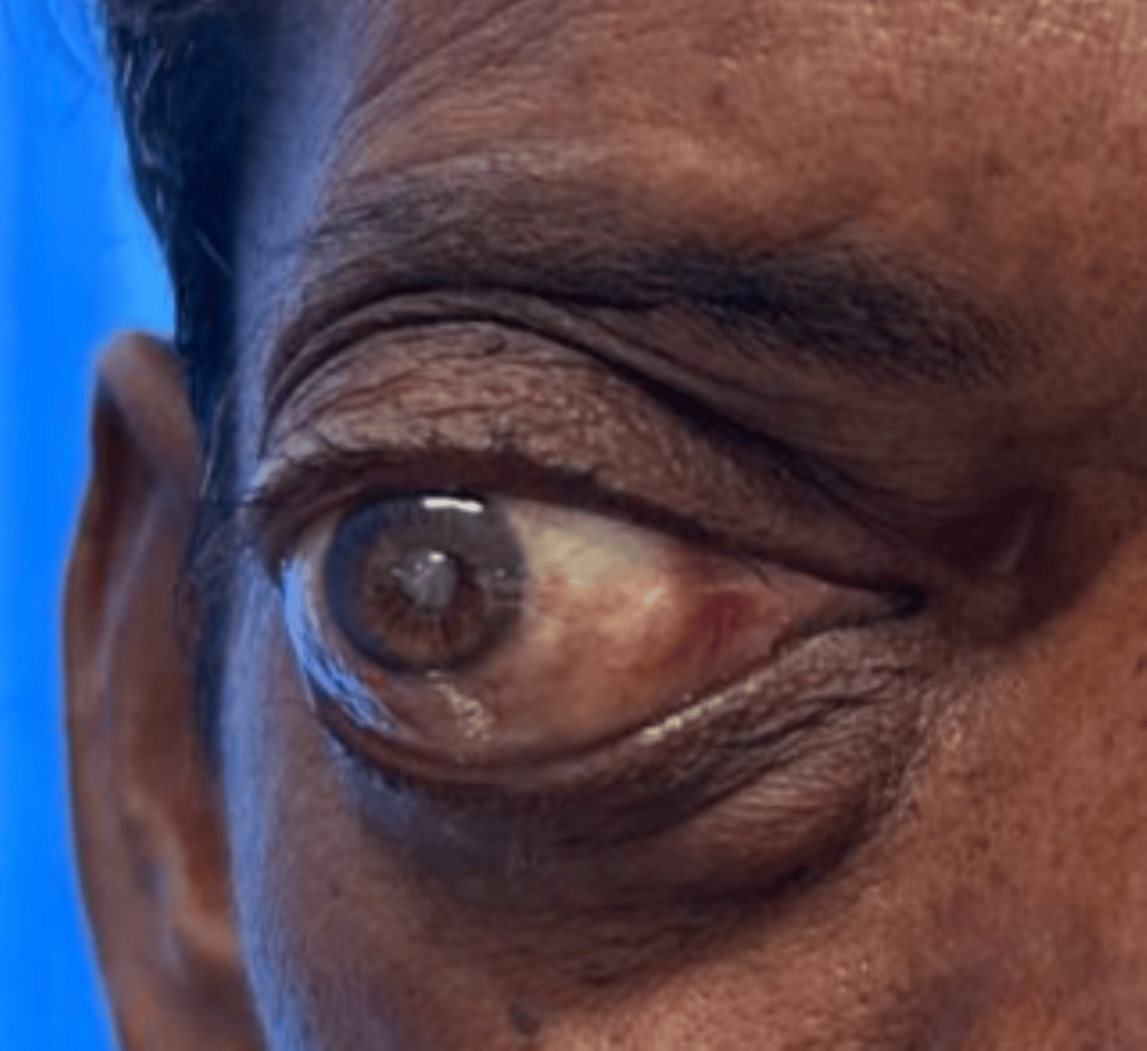
Clinical picture showing abaxial proptosis (down and out) of the right eye.

**Figure 2:**
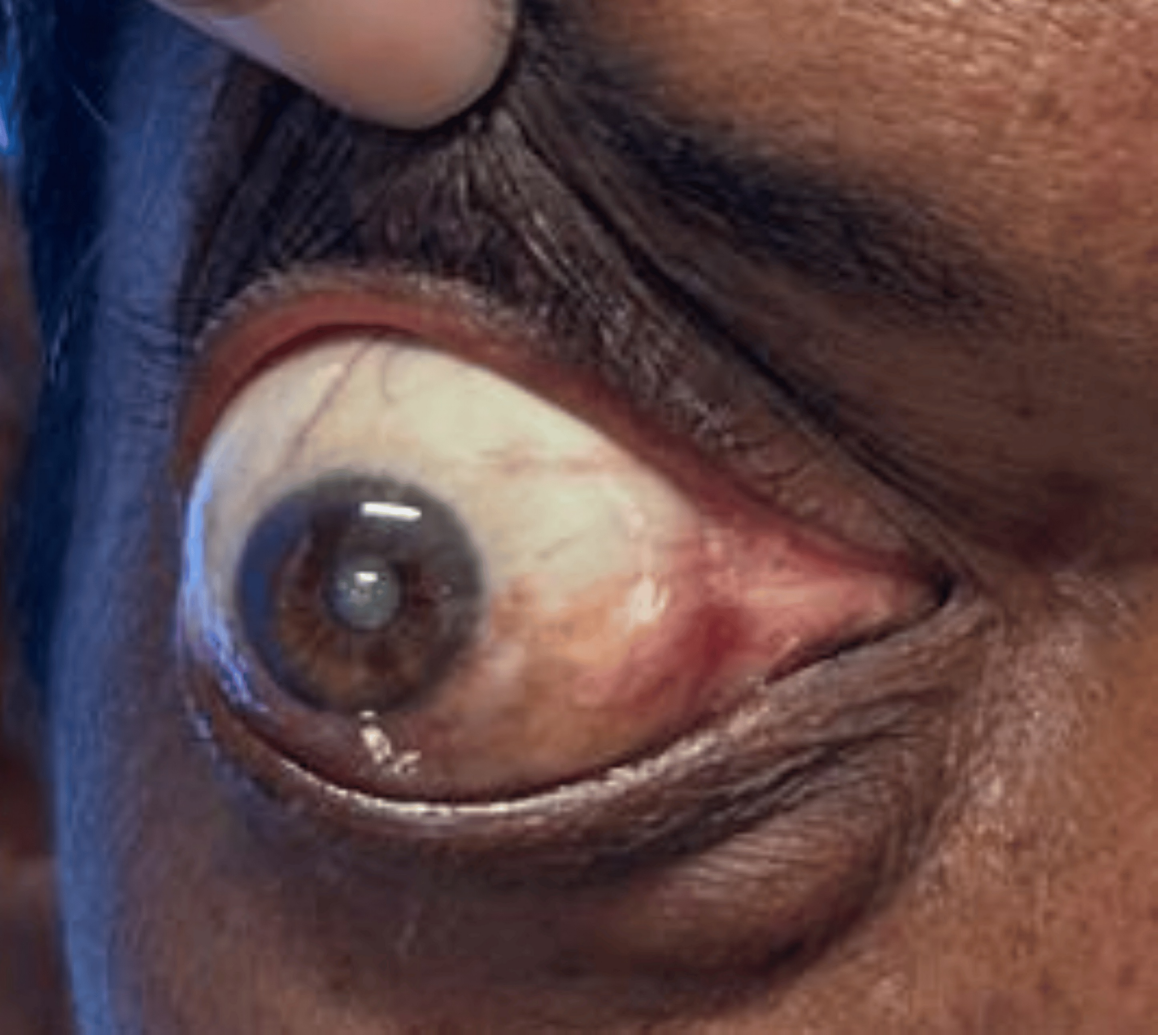
Clinical picture showing abaxial proptosis (down and out) of the right eye.

On examination, there was abaxial proptosis (down and out) of the RE (Figures 1, 2) with mild restriction of extraocular movements in all gazes. Visual acuity in the RE was hand movements close to the face, with accurate projection of rays in all quadrants. The amount of proptosis did not vary with the head-down position or Valsalva maneuver. Exophthalmometry measured 26 mm in the RE. Diffuse conjunctival congestion and mature cataract were noted. Both direct and indirect light reflexes were present in the RE. B-scan ultrasonography showed no evidence of retinal detachment or posterior vitreous detachment. The left eye (LE) examination showed normal anterior and posterior segment findings. Visual acuity in the LE was 6/6.

**Figure 3:**
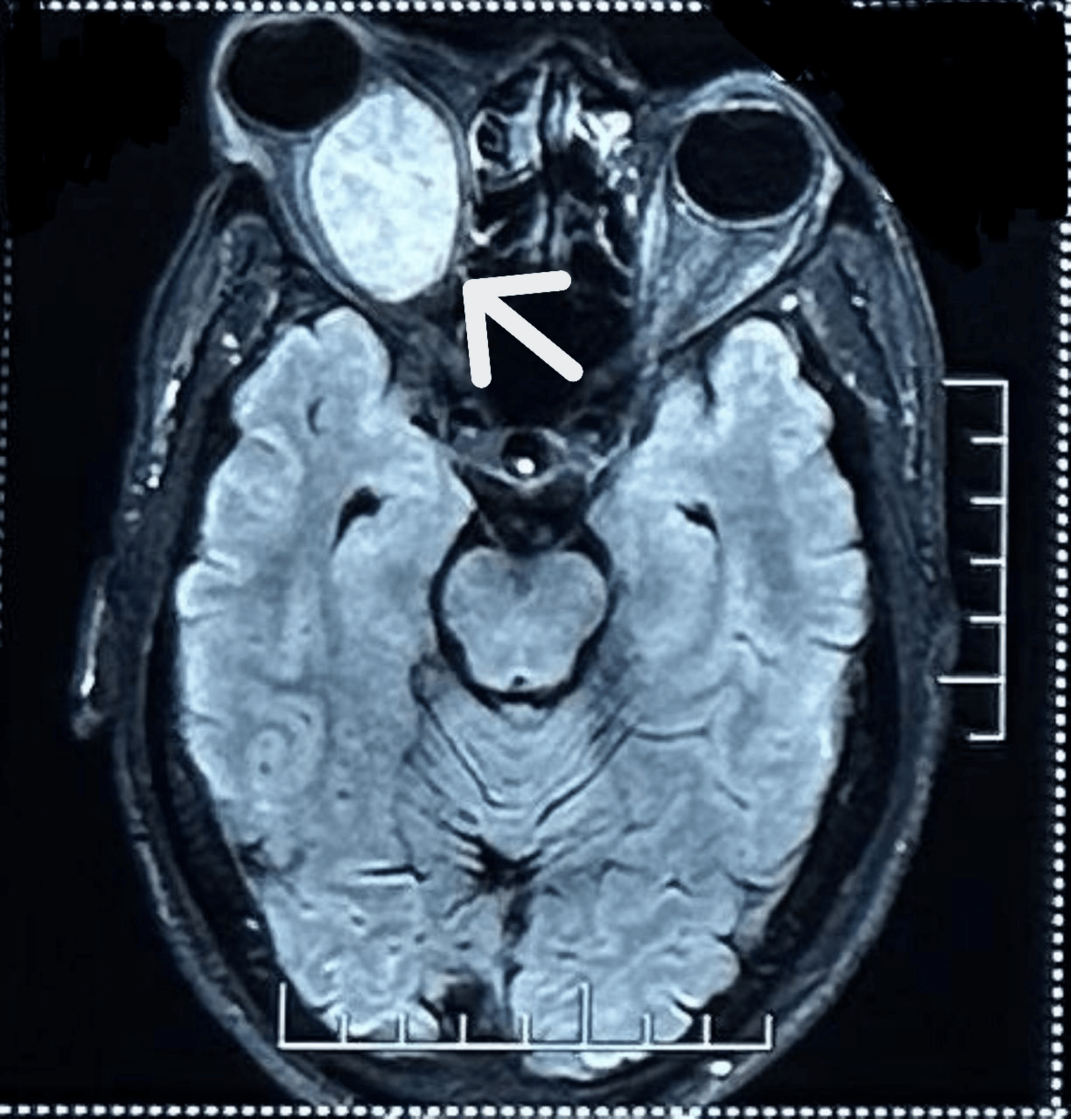
Magnetic resonance imaging of the brain and orbits revealing a well-defined altered lesion measuring 3.2 × 2.4 × 3.4 cm in the intraconal compartment of the right retrobulbar region.

Magnetic resonance imaging (MRI) of the brain and orbits (T2-weighted with contrast) revealed a well-defined intraconal retrobulbar lesion in the right orbit, measuring 3.2 × 2.4 × 3.4 cm, compressing and laterally displacing the optic nerve, which appeared attenuated in caliber (Figure 3).

**Figure 4:**
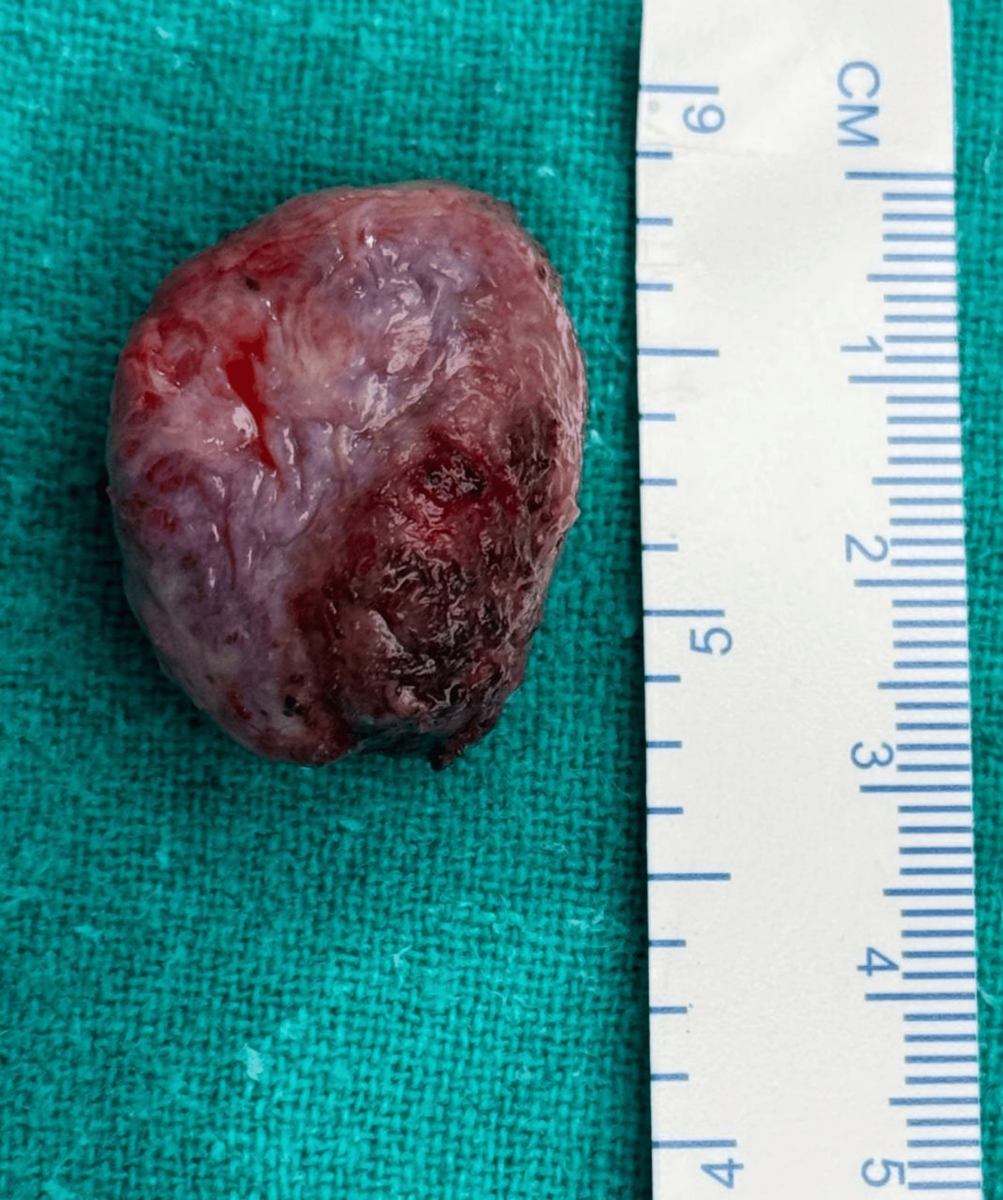
Gross examination showing a single, lobular, gray white to gray brown, soft-tissue piece measuring 3.1 × 2.5 × 1.5 cm in size.

The patient underwent endoscopic transnasal excision of the mass under general anesthesia. Bilateral nasal cavities were packed with adrenaline-soaked patties. A right middle turbinectomy was performed, and the sphenoid ostium was identified. The floor of the sphenoid sinus was removed using a Kerrison punch. The medial orbital wall was opened to expose the orbital contents. The medial and inferior rectus muscles were visualized, and a vascular, encapsulated intraconal mass was seen below them. The mass was dissected circumferentially and excised in toto after cauterization. (Figure 4) The defect was reconstructed using a fibroid flap and tissue glue, followed by bilateral nasal packing with Merocel.

**Figure 5:**
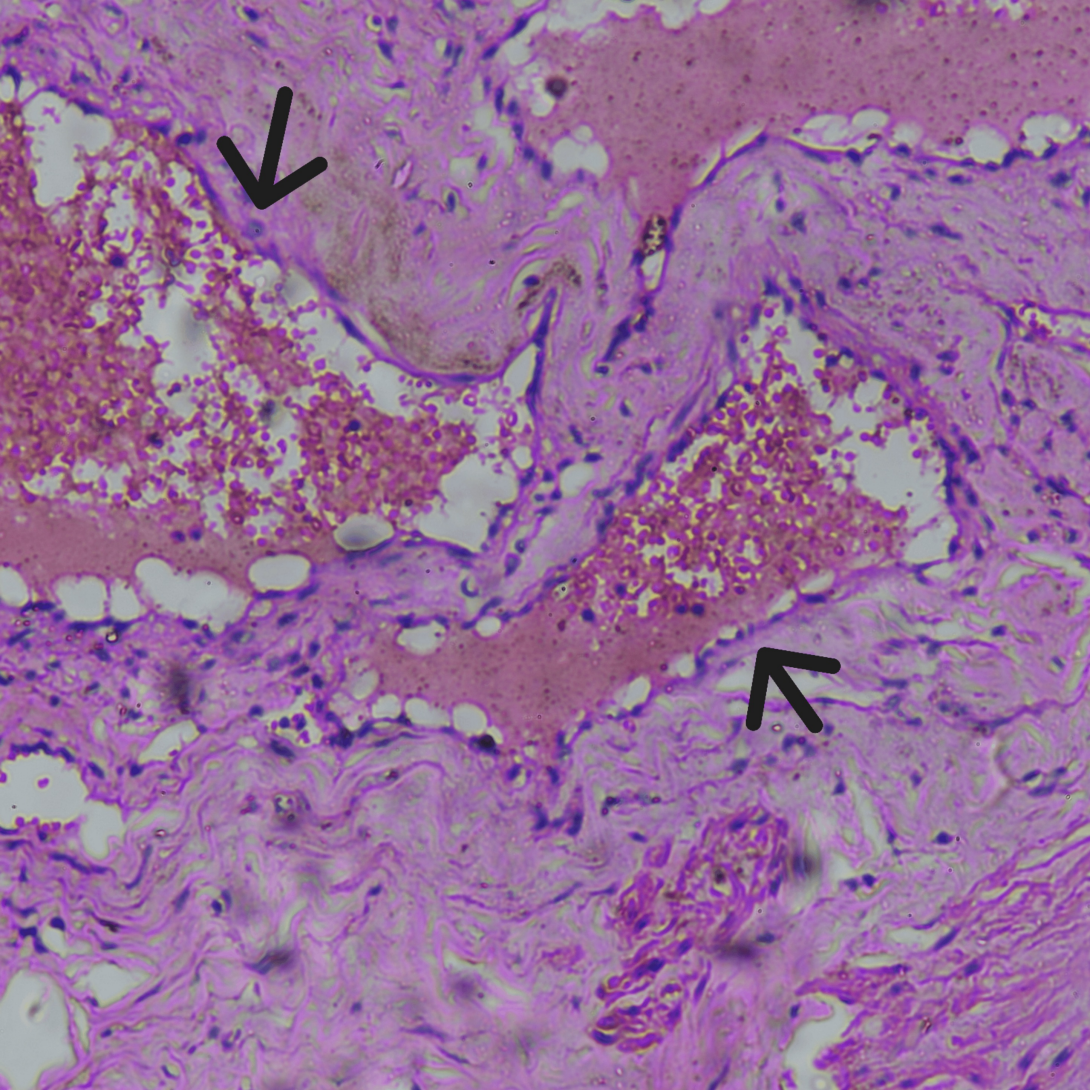
Histopathology of the tissue using hematoxylin and eosin stain under 10×2 magnification showing an uncapsulated tumor with back to back arranged multiple, large calliber, thin-walled, blood-filled channels (black arrow) with scant connective tissue stroma between vessels.

Histopathological examination confirmed the diagnosis of cavernous hemangioma (Figure 5).

**Figure 6:**
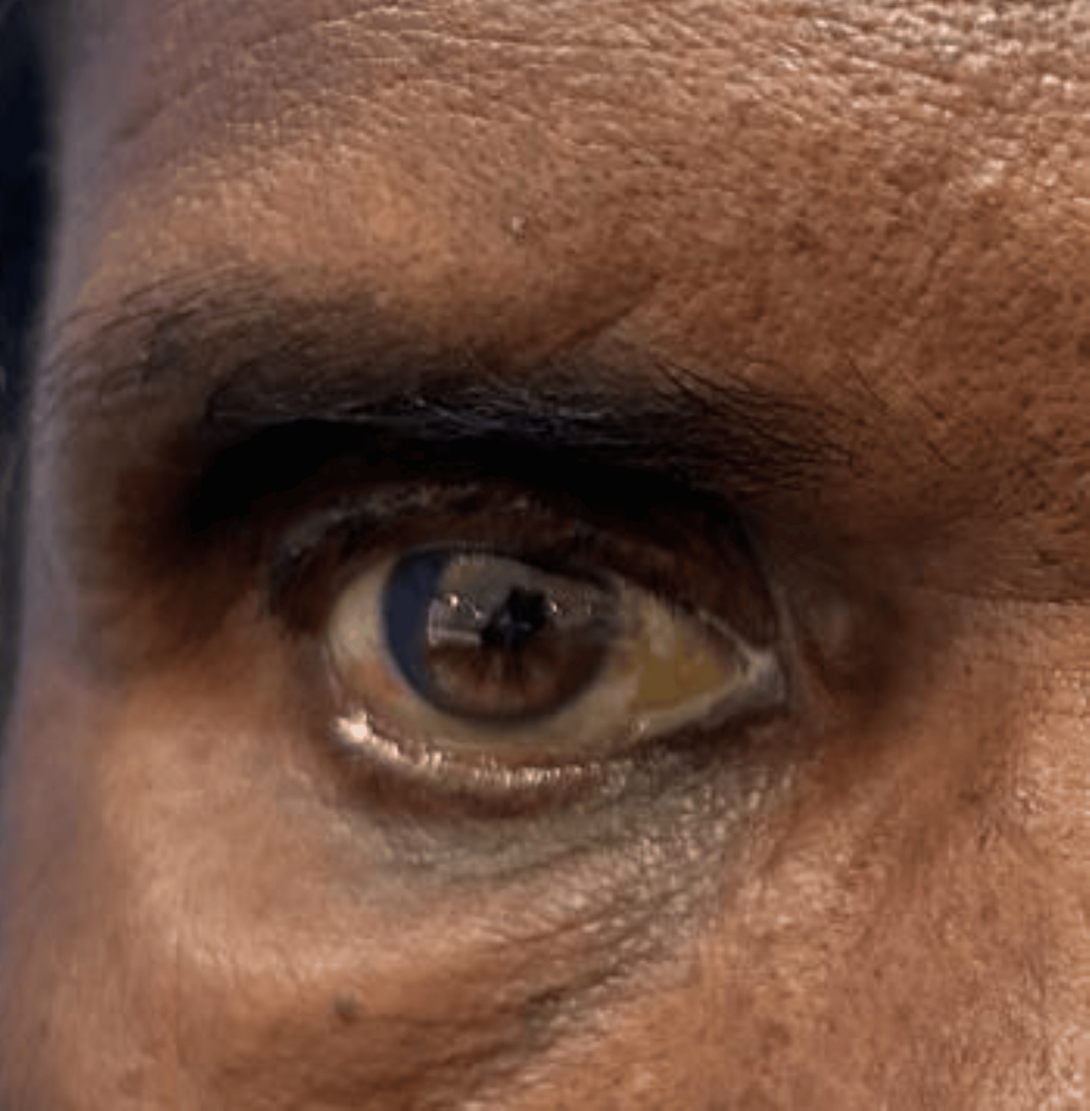
Final clinical image after excision of the right orbital mass and mature cataract extraction of the right eye.

The postoperative course was uneventful, with a reduction in proptosis from 26 mm to 22 mm (Figure 6). Visual acuity remained at hand movements close to the face initially. After RE small-incision cataract surgery, vision improved to 6/60. Following refractive correction using -0.50 dsp and -1.50 dcyl at 40 degrees, the final visual acuity attained was 6/18. Optical coherence tomography of the macula of the RE revealed foveal thinning (181 μm), explaining the suboptimal visual recovery. Macular thickness in the LE was 244 μm.

## Discussion

Cavernous hemangioma of the orbit is the most common benign orbital lesion in adults and the third most frequent diagnosis after lymphoid tumors and idiopathic orbital inflammation [5]. These lesions are considered low-flow, non-distensible venous malformations consisting of septated, thrombosed venous spaces surrounded by a dense fibrous capsule.

Cavernous hemangioma of the orbit typically presents in the third to fifth decade of life as unilateral, painless, progressive axial proptosis [6]. A decrease in vision due to optic nerve compression is usually subtle. Women may experience lesion enlargement during pregnancy due to hormonal influence.

Imaging plays a pivotal role in diagnosis. On ultrasound, cavernous hemangioma of the orbit appears as a well-defined, homogenous, hyperechogenic lesion with low-flow vascular channels on color Doppler. On computed tomography, it is a sharply defined, encapsulated, hyperdense lesion that enhances mildly after contrast administration. MRI remains the investigation of choice, showing a homogenous, well-circumscribed mass that is isointense to muscle on T1-weighted and hyperintense on T2-weighted sequences, often compressing the optic nerve [7].

Surgical excision is indicated in symptomatic cases. The choice of approach depends on tumor location. Traditionally, lateral orbitotomy is used for lateral intraconal lesions, while medial and inferomedial lesions can be approached via the endoscopic transnasal route. Lenzi et al. [8] and Bleier et al. [9] have reported favorable outcomes using the transnasal endoscopic approach for intraconal cavernous hemangiomas in the medial orbit. However, literature remains limited, warranting further case-based evidence.

In our case, the location of the tumor was medial in the intraconal compartment of the right retrobulbar region, leading to abaxial proptosis. The endoscopic transnasal approach provided excellent visualization, precise dissection, minimal morbidity, and satisfactory cosmetic and functional outcomes.

## Conclusions

Intraconal cavernous hemangioma most commonly produces axial proptosis; however, eccentric intraconal lesions, particularly those abutting the orbital wall or located asymmetrically, may produce abaxial proptosis. This case highlights the importance of a multidisciplinary approach involving an oculoplastic surgeon, a neurosurgeon, a radiologist, and a histopathologist in the management of proptosis. Good outcomes can be anticipated in such cases if managed appropriately, as in our case. This case suggests that the transnasal endoscopic approach is a safe, minimally invasive, and efficient approach for the removal of retro-orbital intraconal tumors of the medial orbit. However, further case-based evidence is warranted to report outcomes using the transnasal endoscopic approach for the removal of medially located lesions.
